# Characterization of the Nucleus Pulposus Progenitor Cells via Spatial Transcriptomics

**DOI:** 10.1002/advs.202303752

**Published:** 2024-02-04

**Authors:** Yu Chen, Long Zhang, Xueqing Shi, Jie Han, Jingyu Chen, Xinya Zhang, Danlin Xie, Zan Li, Xing Niu, Lijie Chen, Chaoyong Yang, Xiujie Sun, Taifeng Zhou, Peiqiang Su, Na Li, Matthew B. Greenblatt, Rongqin Ke, Jianming Huang, Zhe‐Sheng Chen, Ren Xu

**Affiliations:** ^1^ The First Affiliated Hospital of Xiamen University‐ICMRS Collaborating Center for Skeletal Stem Cells State Key Laboratory of Cellular Stress Biology Faculty of Medicine and Life Sciences School of Medicine Xiamen University Xiamen 361102 China; ^2^ Xiamen Key Laboratory of Regeneration Medicine Fujian Provincial Key Laboratory of Organ and Tissue Regeneration School of Medicine Xiamen University Xiamen 361102 China; ^3^ Gene Denovo Biotechnology Co Guangzhou 510006 China; ^4^ School of Medicine and School of Biomedical Sciences Huaqiao University Quanzhou 362000 China; ^5^ School of Life Sciences Westlake University Hangzhou 310030 China; ^6^ China Medical University Shenyang Liaoning 110122 China; ^7^ Department of Chemical Biology College of Chemistry and Chemical Engineering Xiamen University Xiamen 361005 China; ^8^ Department of Obstetrics and Gynecology School of Medicine Xiang'an Hospital of Xiamen University Xiamen University Xiamen 361102 China; ^9^ Department of Spine Surgery Guangdong Provincial Key Laboratory of Orthopedics and Traumatology The First Affiliated Hospital of Sun Yat‐sen University Guangzhou 510080 China; ^10^ Department of Pathology and Laboratory Medicine Weill Cornell Medical College New York NY 10065 USA; ^11^ Research Division Hospital for Special Surgery New York NY 10065 USA; ^12^ Department of Orthopedics Chengong Hospital (the 73th Group Military Hospital of People's Liberation Army) affiliated to Xiamen University Xiamen 361000 China; ^13^ College of Pharmacy and Health Sciences St. John's University New York NY 11439 USA

**Keywords:** nucleus pulposus, spatial transcriptomics (ST), stem/progenitor cell, tervertebral disc degeneration, Tie2

## Abstract

Loss of refreshment in nucleus pulposus (NP) cellularity leads to intervertebral disc (IVD) degeneration. Nevertheless, the cellular sequence of NP cell differentiation remains unclear, although an increasing body of literature has identified markers of NP progenitor cells (NPPCs). Notably, due to their fragility, the physical enrichment of NP‐derived cells has limited conventional transcriptomic approaches in multiple studies. To overcome this limitation, a spatially resolved transcriptional atlas of the mouse IVD is generated via the 10x Genomics Visium platform dividing NP spots into two clusters. Based on this, most reported NPPC‐markers, including Cathepsin K (Ctsk), are rare and predominantly located within the NP‐outer subset. Cell lineage tracing further evidence that a small number of Ctsk‐expressing cells generate the entire adult NP tissue. In contrast, Tie2, which has long suggested labeling NPPCs, is actually neither expressed in NP subsets nor labels NPPCs and their descendants in mouse models; consistent with this, an in situ sequencing (ISS) analysis validated the absence of Tie2 in NP tissue. Similarly, no *Tie2*‐cre‐mediated labeling of NPPCs is observed in an IVD degenerative mouse model. Altogether, in this study, the first spatial transcriptomic map of the IVD is established, thereby providing a public resource for bone biology.

## Graphical abstract

The first spatial transcriptomic map of the intervertebral disc (IVD) is established via the 10x Genomics Visium platform. Combined with cell lineage tracing, spatial transcriptome analysis identified nucleus pulposus stem/progenitor cell (NPPC) markers. Tie2 is actually absent in mouse NP at postnatal stage. Together, this spatially resolved transcriptional atlas of the IVD has provided a public resource for bone biology.

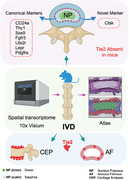

## 1 Introduction

Anatomically, the adult IVD is composed of three distinct yet interdependent units: the central viscous NP, the outer fibrillary annulus fibrosus (AF), and the thin hyaline cartilaginous endplates (CEP) between the adjacent vertebral bodies. Each of these structures displays signature functional and phenotypic characteristics maintained by distinct cellular populations. Compared to the AF and CEP, embryonic notochord‐derived NP tissue is a key determinant of IVD homeostasis via its function to provide positive swelling pressure or turgor.^[^ ^1^ ^]^ Similar to articular cartilage, avascular and abneural NP tissue is also particularly prone to dysfunction with aging. The loss of notochord‐derived NP cells has been nominated as a key early driver of IVD degeneration (IDD).^[^ ^2^ ^]^ As a common musculoskeletal disorder, IDD frequently leads to chronic low back pain, resulting in disability, substantial morbidity, and healthcare expenditures.^[^ ^3^ ^]^ Lower back pain is the leading cause of lost productivity globally and the leading cause of loss of health span in 126 countries and costs an estimated $50–$100 billion a year in surgical expenses in the United States.^[^ ^4^ ^]^ Given this unmet clinical need, basic and clinical research on low back pain has increasingly become an area of intense interest.^[^ ^5^ ^]^ Clinically, surgical management of IDD focuses primarily on spine fusion and cannot restore the damaged IVD.^[^ ^6^ ^]^ Therefore, there is an urgent need to develop medical therapies that address IVD biology and restore NP cellularity for the treatment of IDD.

NP tissue includes cells that behave stem cell‐like properties, such as multipotency and clonogenicity, in vitro.^[^ ^7^ ^]^ Given this, cell therapy for NP replacement has been an area of interest as it offers the potential to regenerate IVD components while being minimally invasive. To achieve this, identifying NPPC markers is a prerequisite for NPPC isolation and clinical translation. Notably, most of the earlier studies in NPPC characterization are solely based on observations in cell culture systems or bulk transcriptional profiling, which hinders in‐depth exploration of these NPPC markers. For example, as a pioneering discovery,^[^ ^8^ ^]^ dual positivity of Tie2 (also known as CD202) and GD2 (ganglioside) has long been suggested to determine the hierarchy of NP stem/progenitor cells in multiple studies across species.^[^ ^9^ ^]^ Nevertheless, the presence and importance of Tie2 in NP tissue remain disputable, partially due to a lack of genetic evidence in vivo. In addition to Tie2, a few recent studies were initiated to discover NPPC populations through conducting cell‐fate mapping in vivo. For instance, cell‐lineage tracing combined with single‐cell RNA sequencing (scRNA‐Seq) identified urotensin II receptor (Uts2r) as a genetic marker targeting postnatal NPPCs with regenerative potential.^[^ ^1^ ^]^

However, scRNA‐Seq analysis is still limited due to the potential loss of cell populations during isolation procedures and the absence of spatial information at the single‐cell level. Now, advanced ST techniques can offer both transcriptomic profiling and information on the positional context of tissue sections, which provides key information for defining cellular phenotypes.^[^ ^10^ ^]^ In this study, we first generated a spatially resolved transcriptome‐atlas of the mouse IVD to visualize the expression pattern of potential NPPC marker genes. Interestingly, most NPPCs including newly identified Ctsk‐expressing cells, are predominantly present in the peripheral region of NP tissues; however, Tie2 does not mark cells contributing to NP architecture in mice.

## 2 Results

### 2.1 The First Spatially Resolved Transcriptome‐Atlas of the IVD

**Figure 1:**
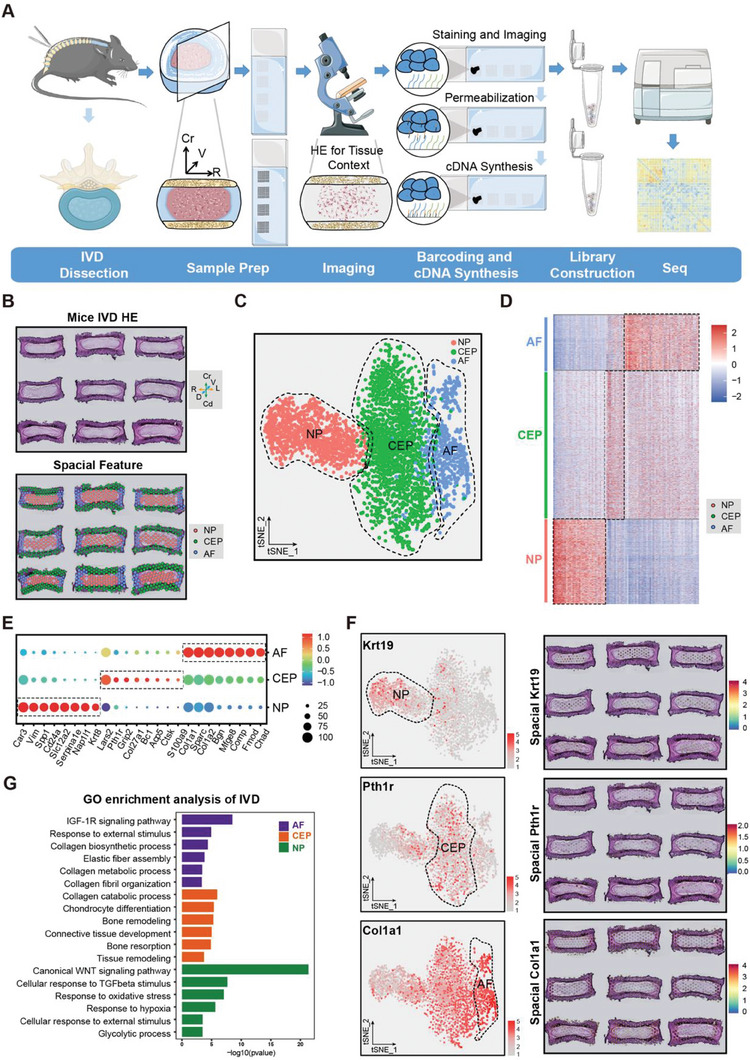
10x Genomics Visium ST analysis of IVD in mice. A) Schematic workflow of the experimental strategy for ST of IVD. B) A spatial feature plot and three clusters (AF, CEP, and NP) were identified. C) The tSNE plots of all cells from ST of 3‐week mice IVD. D) Heatmap showing the scaled expression of differentially expressed genes for each cluster in IVD. E) Dot plots show the mean expression of genes among the three subclusters. Dot size indicates the percentage of cells in subclusters with detected expression. The color changed from grey to blue as the gene expression levels increased. F) The tSNE plots and spatial feature plots of novel genes in each cluster. G) Representative analysis of GO categories showing different functions for AF, NP, and CEP clusters.

NP tissues are extraordinarily fragile and gelatinous and have greatly limited cell enrichment according to conventional transcriptomic approaches, including single‐cell sequencing. The ST can map the location of specific transcripts in tissues and identify the expression locations of specific genes, thus enabling a more comprehensive study of tissues at the molecular level. Therefore, we employed the 10x Genomics Visium platform^[^ ^11^ ^]^ to generate a spatially resolved transcriptomic landscape of the mouse postnatal IVD (**Figure** **1**A). To obtain adequate IVD‐derived spots for downstream characterization, we profiled spatial gene expression in 39 coronal sections of 4 chips of IVD tissue from 3‐week‐old mice (Figure 1B; Figure S1A–C, Supporting Information). These IVD regions comprised a total of 3860 spots with a mean depth of 4888966 reads, which corresponded to a median of 566 unique molecular identifiers (UMIs) and a median of 310 genes per spot. Based on the expression patterns of the spots, we defined the NP, AF, and CEP regions within the IVD sections using Seurat, and found that the location of each of these regions of interest was consistent with the location of these IVD components in a serial hematoxylin and eosin (H&E) stained section (Figure 1B,C). In parallel, t‐distributed stochastic neighbor embedding (tSNE) of cell type clustering further showed the similarity of 3 major cell populations derived from the IVD area seen in Seurat: NP cells (key feature genes: Car3,^[^ ^12^ ^]^ Krt19,^[^ ^13^ ^]^ T,^[^ ^14^ ^]^ Cd24a^[^ ^15^ ^]^ and Vim^[^ ^16^ ^]^), AF cells (key feature genes: Col1a1,^[^ ^17^ ^]^ Col2a1,^[^ ^18^ ^]^ Comp,^[^ ^19^ ^]^ Bgn,^[^ ^20^ ^]^ and Sparc^[^ ^21^ ^]^) and CEP cells (key feature genes: Grip2,^[^ ^22^ ^]^ Col27a1,^[^ ^23^ ^]^ S100a9,^[^ ^24^ ^]^ Ibsp,^[^ ^25^ ^]^ and Pth1r^[^ ^5d^ ^]^) (Figure 1D–F; Figure S1D and Table S1, Supporting Information). Additionally, we found new key feature genes, Slc12a2, Myh1, and ACP5, in the NP, AF, and CEP clusters, respectively (Figure S1E, Supporting Information). Consistent with this, Gene Ontology (GO) term enrichment analysis revealed the distinctive characteristics in each region of the IVD atlas population, such as “cellular response to transforming growth factor beta (TGFbeta) stimulus” in the NP cluster, “collagen biosynthetic process” in the AF cluster and “bone remodeling” in the CEP cluster (Figure 1G; Table S2, Supporting Information). A similar characterization of the NP, AF, and CEP subsets was also performed via reactome analysis (Figure S1F; Table S3, Supporting Information).

**Figure 2:**
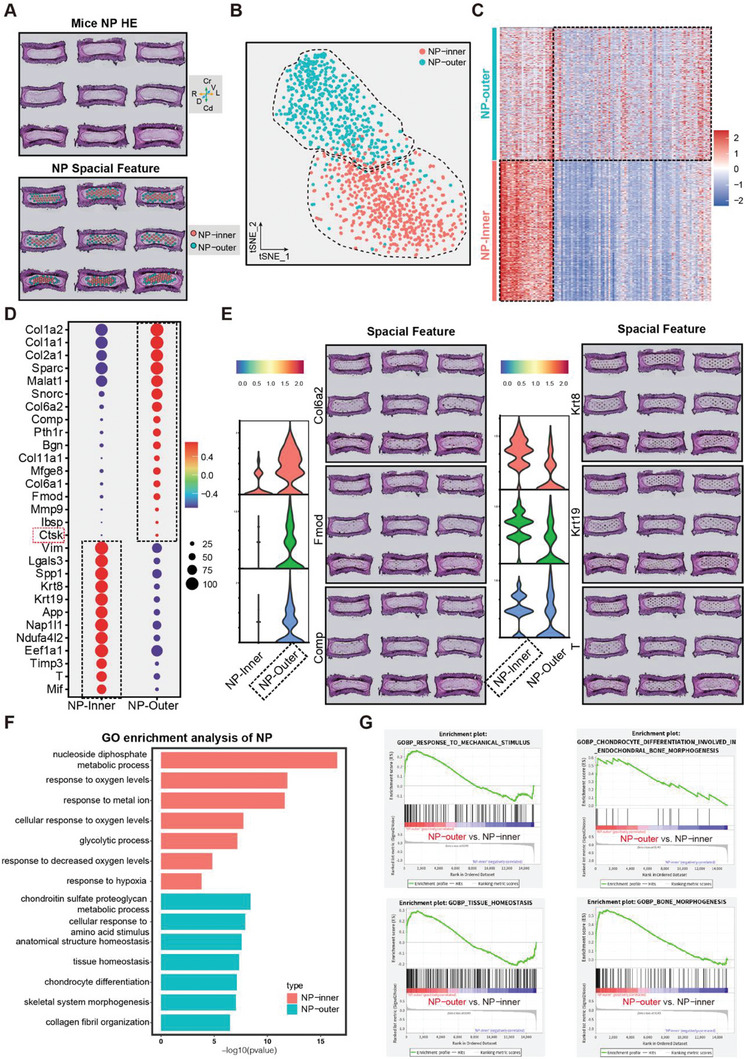
10x Genomics Visium ST analysis of NP in mice. A) A spatial feature plot and two clusters (NP‐inner and NP‐outer) were identified. B) The tSNE plots analysis shows two clusters of NP cells labeled with different colors. C) Heatmap showing the scaled expression of differentially expressed genes for each cluster in NP. D) Dot plots showing the mean expression of genes among the two subclusters in NP. Dot size indicates the percentage of cells in subclusters with detected expression. The color changed from gray to blue as the gene expression levels increased. E) The tSNE plots and spatial feature plots of representative genes in each cluster. F) Representative analysis of GO categories showing different functions for NP‐inner and NP‐outer clusters. G) GSEA showing the enrichment of pathways between NP‐inner and NP‐outer clusters.

To understand the components of the NP region in ST, we performed dimensionality reduction and clustering to divide the NP spots into two subsets: NP cluster‐0 and NP cluster‐1 (**Figure** **2**A–C; Figure S2A, Supporting Information). Notably, the chondrocyte‐related markers, such as Col6a2,^[^ ^26^ ^]^ Fmod^[^ ^27^ ^]^ and Comp,^[^ ^19^ ^]^ were dominantly expressed in NP cluster‐1; moreover, NP cluster‐0 was enriched in the high levels of classical notochord/NP genes, such as Krt8, Krt19 and T (Figure 2D; Figure S2B and Table S4, Supporting Information). Interestingly, spatial visualization further indicated that the spots in NP cluster‐1 were mostly distributed around the NP periphery, while NP cluster‐0 mainly covered the central NP area (Figure 2D,E). Based on these findings, we renamed these two clusters NP‐outer cells (NP cluster‐1) and NP‐inner cells (NP cluster‐0), which responded to specific biological properties and signaling pathways, respectively, as evidenced by the GO term (Figure 2F; Table S5, Supporting Information) and Kyoto Encyclopedia of Genes and Genomes (KEGG) analyses (Figure S2C; Table S6, Supporting Information). Gene set enrichment analysis (GSEA) further showed that the NP‐outer cluster was enriched for the tissue homeostasis, response to mechanical stimulus, chondrocyte differentiation, and bone morphogenesis, which play vital roles in modulating NP homeostasis, in line with the results of the GO analyses (Figure 2G).

**Figure 3:**
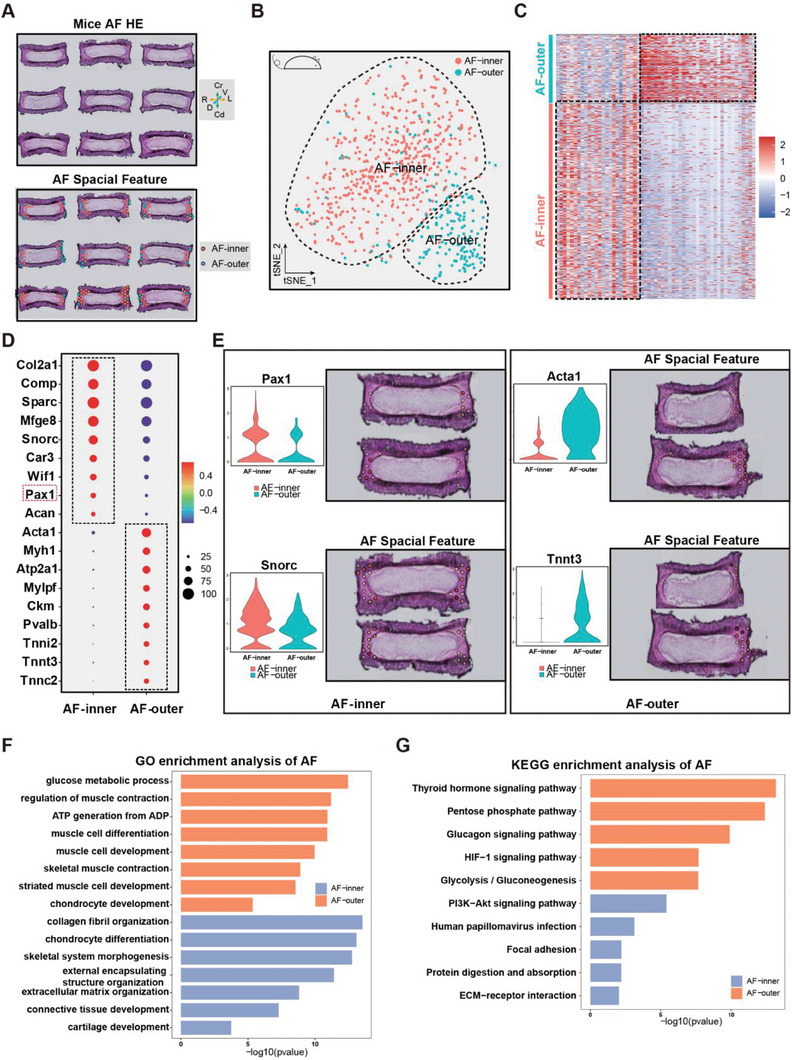
Spatial transcriptomic analysis of AF in mice. A) A spatial feature plot and two clusters (AF‐inner and AF‐outer) were identified. B) The tSNE plots analysis shows two clusters of NP cells labeled with different colors. C) Heatmap showing the scaled expression of differentially expressed genes for each cluster in AF. D) Dot plots showing the mean expression of genes among the two subclusters in two AF clusters. Dot size indicates the percentage of cells in subclusters with detected expression. The color changed from grey to blue as the gene expression levels increased. E) The tSNE plots and spatial feature plots of representative genes in each cluster. F) Representative analysis of GO categories showing different functions for AF‐inner and AF‐outer clusters. G) KEGG analysis of the DEGs showing the enriched signaling pathways in AF.

Like in the NP clustering, the AF cells were further subdivided into AF‐inner and AF‐outer clusters, which anatomically corresponded to the inner AF and outer AF, respectively (**Figure** **3**A,B; Table S7, Supporting Information). The AF‐related marker gene Pax1 was dominantly expressed in the AF‐inner cluster, along with Comp and Snorc.^[^ ^28^ ^]^ The AF‐outer cluster was enriched in the collagenous fiber genes, Acta1,^[^ ^29^ ^]^ Tnnt3^[^ ^30^ ^]^ and Myh1^[^ ^31^ ^]^ (Figure 3C–E; Figure S3A, Supporting Information). Consistent with this, the AF‐inner cells were enriched in terms associated with collagen fibril organization, whereas the AF‐outer cells were enriched in glucose metabolic processes according to the GO term analysis (Figure 3F; Table S8, Supporting Information). Similarly, the KEGG analysis further demonstrated that the ECM‐receptor interaction and the PI3K‐Akt signaling pathway were essential for the AF‐inner cluster, while the pentose phosphate pathway and glucagon signaling pathway mainly responded to the AF‐outer cluster (Figure 3G; Table S9, Supporting Information). Taken together, in line with the anatomical structure, these analyses in ST provided the first spatially resolved transcriptome‐atlas of the IVD.

### 2.2 Evaluation of Proposed NPPC Markers in the Mouse IVD Using a Spatially Resolved Transcriptome Atlas

**Figure 4:**
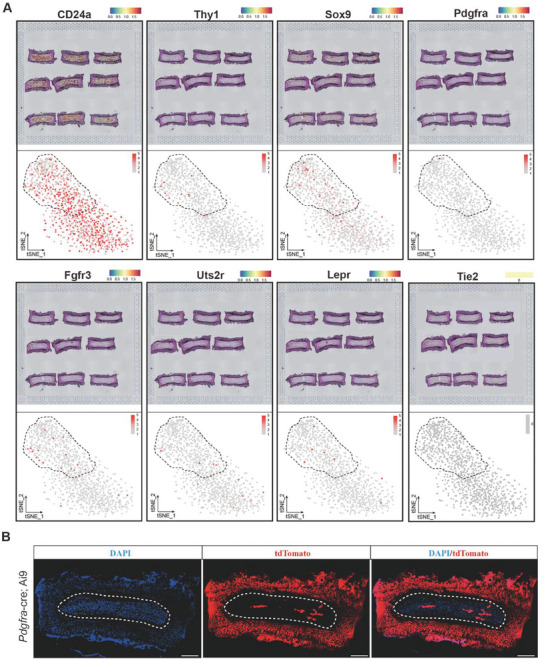
Evaluation of proposed NPPC markers using a spatially resolved transcriptome‐atlas of mouse IVD. A) Spatial distribution with expression intensity and tSNE plots analysis of various markers on the same ST of mouse IVD (CD24a, Thy1, Fgfr3, Lepr, Pdgfra, Uts2r, Sox9, and Tie2). B) Representative images of lumbar spine sections from 3w *Pdgfra‐Cre; Ai9* mice (red) stained for DAPI. A White dashed line surrounded NP region. Scale bars, 200 µm (n = 4 mice per group).

Recently, numerous marker genes have been proposed to define postnatal NPPCs in mouse and human studies (including CD24a,^[^ ^15^ ^]^ CD90 (Thy1),^[^ ^32^ ^]^ Fgfr3,^[^ ^7a^ ^]^ Lepr,^[^ ^7b^ ^]^ Pdgfra (CD140a),^[^ ^33^ ^]^ Uts2r,^[^ ^1^ ^]^ Sox9,^[^ ^34^ ^]^ and Tie2^[^ ^8^ ^]^). To comprehensively compare these NPPC markers head‐to‐head, we examined their local distribution and expression intensity using the ST atlas of the mouse IVD described above. We found that CD24a and Sox9 were widely expressed within the IVD region (**Figure** **4**A; Figure S4A, Supporting Information). Interestingly, the recently identified NPPC markers (Fgfr3, Uts2r, and Lepr), which have been validated through cell‐lineage tracing in vivo, were all rare and found to be highly present in the peripheral regions of the NP in the ST analysis (Figure 4A; Figure S4A, Supporting Information). Consistent with these findings, Pdgfra and CD90,^[^ ^35^ ^]^ which were found to characterize adult NPPCs via human single‐cell transcriptional profiling, were also found to be preferentially expressed in the peripheral region of the total NP structure in the ST analysis (Figure 4A). *Pdgfra‐cre* driven cell lineage tracing demonstrated that a few peripheral Pdgfra‐expressing NPPCs generated the outer and inner structures of the NP (Figure 4B).

**Figure 5:**
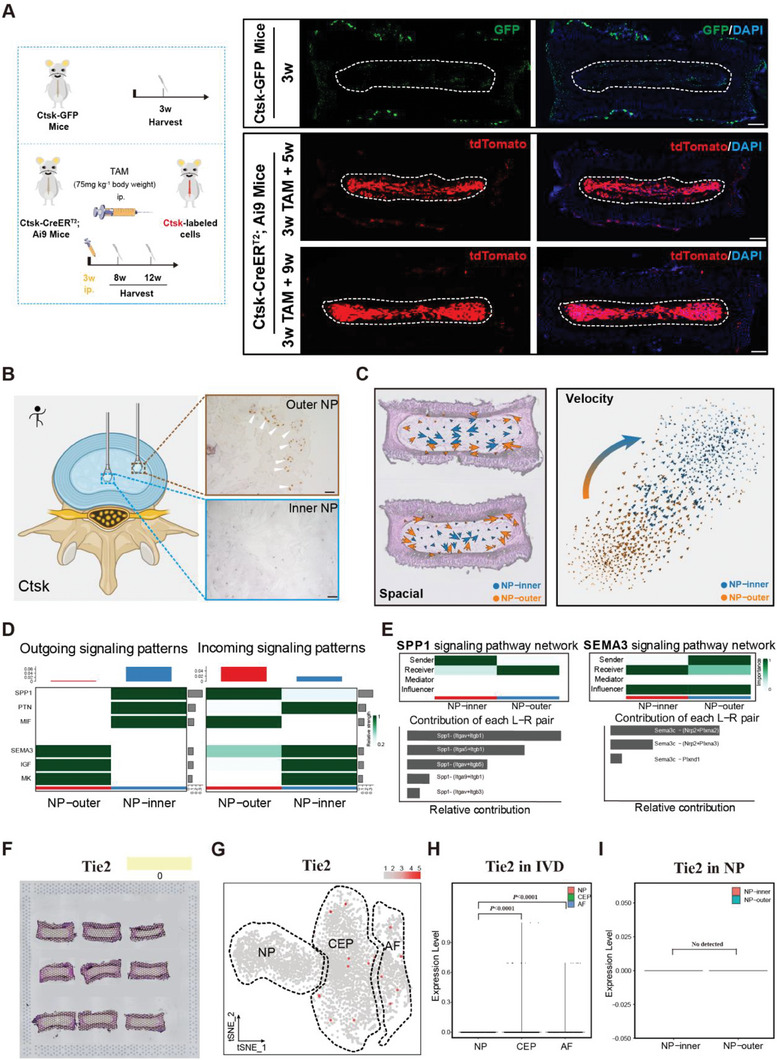
Ctsk+ NP cells contribute to the growth of NP in adult stage of mice. A) Schematic workflow of the experimental strategy. *Ctsk‐CreER^T2^; Ai9* mice were injected with TAM at 3w and the cells were all traced for 8w and 12w. Representative immunofluorescence imaging of *Ctsk‐GFP* cells (green) and *Ctsk‐CreER^T2^; Ai9*+ cells (red) (n = 5 mice per group). B) Representative IHC images of Ctsk in human NP sections at the outer region and inner region. Scale bar, 200 µm. C) Image of velocity depicting the potential trajectories of NP subpopulations. Source data are provided as a Source Data file. D) Heatmaps of signals contributing mostly to outgoing or incoming signaling of NP cell groups. E) Heatmap shows the role (sender, receiver, mediator, or influencer) of each cell group in SPP1/SEMA3 signaling. Diagram of ligand‐receptor pair contribution to SPP1/SEMA3 signaling pathway. F) A spatial feature plot of Tie2 in the NP region was identified. G) The tSNE plots analysis shows Tie2 in two clusters (AF and CEP), but not in NP cluster. H) Violin plots showing the expression levels of Tie2 in IVD. I) Violin plots showing the expression levels of Tie2 in NP.

Notably, as an essential marker of periosteal stem cells, tendon progenitors,^[^ ^36^ ^]^ and mature osteoclasts, Ctsk was also present at the periphery of the NP, AF, and CEP at the postnatal stage (Figure S5G, Supporting Information). However, bioinformatic analysis of the Ctsk+ cells in these three regions revealed heterogeneity. GSEA revealed that Ctsk in the NP was enriched for genes related to developmental cell growth, tissue remodeling, and tissue regeneration. Ctsk in the AF was enriched for connective tissue development and the cellular response to mechanical stimuli. Ctsk in CEP was enriched for osteoclast differentiation and bone resorption (Figure S4B, Supporting Information). In contrast to Ctsk+ CEP cells and Ctsk+ AF cells, Ctsk+ NP cells were derived from the notochord. We used Sox10‐Cre;Ai9 mice, which can mark notochord‐tracing cells, to construct Ctsk+ NP cells (Abcam, ab19027, Cambridge, UK), which were costained with Sox10+ lineage cells by immunofluorescence (Figure S5H, Supporting Information). Focusing on the NP region, the transient expression of Ctsk in *Ctsk‐GFP* mice and Ctsk ‐lineage cells in *Ctsk‐creERT2; Ai9* mice after 24h tracing were both in line with the results of the ST analysis. Ctsk+ cells were preferentially expressed at the NP periphery (**Figure** **5**A; Figure S5A–I, Supporting Information).

Consistent with these findings, a high expression level of CTSK was also detected in the peripheral region of human NP tissue (Figure 5B). Next, we mapped the fate of Ctsk+ NPPCs in vivo using *Ctsk‐creER^T2^; Ai9* as a tracer tool and showed that a small number of Ctsk+ cells in the peripheral region contributed to the whole morphological profile of the NP (Figure 5A). Hence, all these observations indicated that NPPCs were preferentially distributed in the peripheral region of the NP and subsequently generated outer and central NP cellularity with IVD maturation, which was further supported by the RNA velocity analysis for the NP subsets in the ST above (Figure 5C; Figure S5B, Supporting Information). To confirm whether Ctsk+ NP cells are the progenitor cells in the NP, we isolated Ctsk+ cells from *Ctsk‐creER^T2^; Ai9* mice using fluorescence‐activated cell sorting (Figure S5D, Supporting Information). NP sphere formation analyses revealed a significantly greater sphere‐forming efficiency with significantly more spheres (Figure S5E, Supporting Information). Oil red staining, Alizarin red staining, and Toluidine blue staining revealed that the Ctsk+ cells had multipotent capabilities (differentiated into various cell lineages, including osteoblasts, adipocytes, and chondrocytes) (Figure S5F, Supporting Information).

To understand the intercellular communication between these two NP subsets, we performed a systematic analysis of cellular communication networks using the Cell Chat package in R (version 4.2.0) and revealed a series of efferent or afferent signaling pathways, including osteopontin (OPN, SPP1), pleiotrophin (PTN), macrophage migration inhibitory factor (MIF), secreted class 3 (Sema3), insulin‐like growth factor (IGF) and midkine (MK) (Figure 5D; Figure S5C, Supporting Information). Among these, Spp1‐(Itgav+Itgb1) was the major ligand‐receptor pair that sends signals from the NP‐inner to the NP‐outer; moreover, the NP‐outer subset was the dominant source of SEMA3 signals targeting the NP‐inner subset mainly via the Sema3c‐(Nrp2+Plxna2) axis (Figure 5E). Consistent with this, in the meningeal region, Spp1‐Itgav intimately interacts with microglia and neurons,^[^ ^37^ ^]^ and Spp1 may largely regulate Itgb1‐mediated smooth muscle cell (SMC)‐fibroblast communication.^[^ ^38^ ^]^

To further elucidate the temporal and spatial dynamics of these postanal NPPC markers, we visualized their expression patterns during embryonic IVD formation in our prior ST database and compared them to those of the current study. Based on these findings, Cd24a, Sox9, Pdgfra, Fgfr3, Lepr, and Ctsk were all expressed at embryonic and postnatal stages, as were canonical notochord markers such as Krt19^[^ ^39^ ^]^ (Figure S6A,B, Supporting Information); however, the expression of Thy1 and Uts2r was detected only until E13.5 after taking shape of IVD (Figure S6C,D, Supporting Information). Notably, unlike other well‐validated NPPC markers, Tie2 expression was only detectable in the AF and CEP but not in the NP region in the ST atlas of mouse IVDs at the postnatal stage (Figure 5F,G), although some weak expression of Tie2 was present around the embryonic notochord. Similar results were confirmed by in‐depth spot mapping and violin plot analysis of the NP region (Figure 5H,I).

### 2.3 Tie2 is not Expressed in Mouse NP Tissue

**Figure 6:**
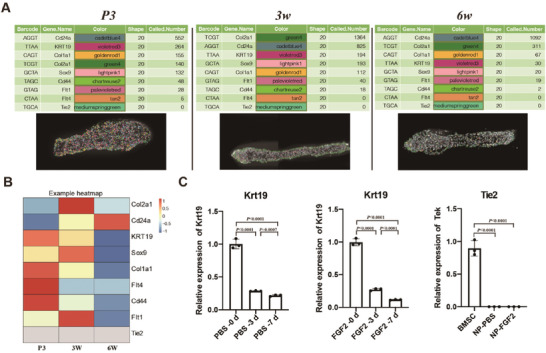
The absence of Tie2 expression in mouse NP tissue. A) Localization of each of the 9 detected barcodes is shown as a symbol on top of a fluorescence microscopy image showing P3, 3w, and 6w NP tissues. Each symbol represents a barcode sequence that is in accord with a specific transcript. Expression profiling of the 9 transcripts in three different stages of NP region (Left images of each stage). Transcripts counted in the NP region were normalized to those in the DAPI‐positive region (Right images of each stage) (n = 6 mice per group). B) The heatmap showed the expression of 9 genes with age. C) qRT‐PCR of NP cells cultured in vitro showed the expression of Krt19 decreased without (with PBS, Left image) or with FGF2 (Middle image) as culture time increased and the expression of Tie2 between groups (with or without FGF2) and positive control group (bone marrow‐derived mesenchymal stem cells, BMSC). d, day.

During the process of NPPC identification, Tie2‐positive NP cells with high self‐renewal ability and multipotency were suggested to constitute the first and most vital NPPC population. However, it remains controversial whether Tie2 is expressed in NP cells within human or mouse IVD tissue has not been determined. Given the absence of Tie2 expression in our ST, we further employed an in situ sequencing (ISS) approach for visualizing of multiple mRNA transcripts in the NP region of mouse IVD sections. In addition to the Tie2 probe, we added other NP cell markers including Krt19 and Col2a1, as positive controls for transcript visualization in the mouse NP at the P3, 3‐week‐old, and 6‐week‐old stages (**Figure** **6**A). As expected, all the selected NP markers were present in the NP region but with different expression intensities during NP maturation (Figure 6A,B). However, Tie2 was still undetectable in the NP region at any stage, although the other candidate NPPC‐markers, such as Cd44 and Flt1, were significantly expressed (Figure 6A,B). Consistent with these findings, the expression level of Tie2 was not detectable by real‐time PCR analysis without or with FGF2 stimulation in cultured NP cells, whereas Krt19 expression was detected under the same conditions as a positive control (Figure 6C). Collectively, these in vivo and in vitro gene expression results suggested that, compared to that of other NPPC markers, Tie2 expression was undetectable in mouse NP cells.

### 2.4 Tie2+ Cells are not a Physiological Source of NPPCs in the Mouse IVD

**Figure 7:**
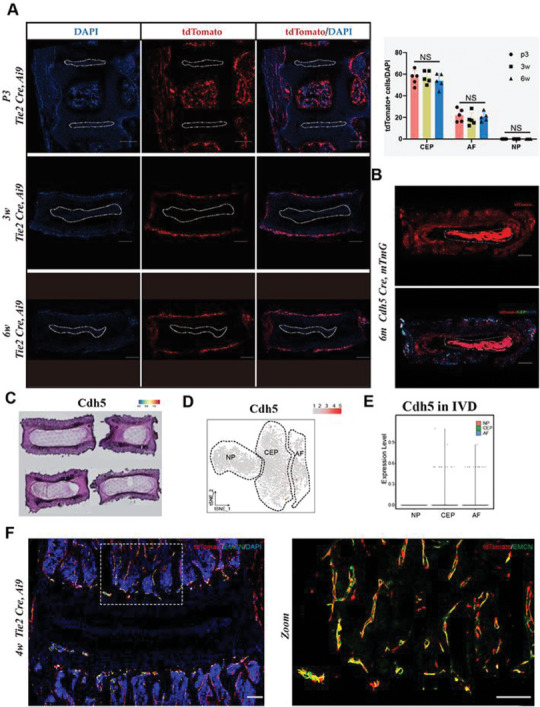
Tie2‐lineage cells are not a physiological source of NP cells. A) Representative images of lumbar spine sections from P3, 3w, and 6w *Tie2‐Cre; Ai9* mice (red) stained for DAPI (n = 5 mice per group). A White dashed line surrounded NP region (Left). Quantification of tdTomato+ cells (Right). B) Representative images of lumbar IVD sections from 6m *Cdh5‐Cre; mTmG* mice (green) stained for DAPI (n = 5 mice per group). C) Spatial distribution of Cdh5 on the ST of mouse IVD. D) The tSNE plots analysis shows Cdh5 in IVD region labeled with different colors. E) Representative violin plot of Cdh5 in 3 clusters. Scale bars, 200 µm. F) Representative images of Tie2 and EMCN staining from 4w *Tie2‐Cre; Ai9* mice (red) stained for DAPI (n = 5 mice per group).

To examine the possibility that Tie2+ cells outside the NP region contribute to the formation of NP tissues as local stem/progenitor cells, we crossed *Tie2‐Cre* mice^[^ ^40^ ^]^ with Ai9 (R26R‐tdTomato) mice^[^ ^41^ ^]^ to generate *Tie2‐Cre; Ai9* mice (here referred to as tdTomato^Tie2^) as a tool for cell‐lineage tracing. Because Tie2 is expressed in virtually all vascular endothelial cells throughout the developmental and adult stages,^[^ ^42^ ^]^ Ai9^Tie2^ cells were found to include vascular endothelium located in the bone marrow, vertebral bones, and periphery of the AF (**Figure** **7**A). However, the tdTomato‐positive area was not observed in any of the NP regions during the postnatal stages of IVD maturation, consistent with the results of the ISS analysis (Figure 7A). Moreover, another endothelial cell tracer driven by *Cdh5‐cre* showed a similar pattern of blood vessel labeling as that observed in tdTomato^Tie2^ mice, indicating that Tie2+ cell populations are mostly equivalent to Cdh5‐positive vascular endothelial cells in the context of the mouse IVD (Figure 7B). Next, we mapped Cdh5‐positive spots back onto the IVD tissue sections in our ST analysis. Both spatial transcriptional visualization and tSNE clustering analysis revealed that, similar to the Tie2 expression pattern, all Cdh5‐occupied spots present in the AF or CEP region contained vascular endothelium (Figure 7C–E). Costaining for Tie2 and vascular markers (CD31 and EMCN) revealed that Tie2 mostly labels vascular endothelial cells but not NP cells (Figure 7F; Figure S7A, Supporting Information). These results further demonstrated that Tie2 mostly labels vascular endothelial cells that cannot contribute to NP cellularity.

### 2.5 Tie2‐Lineage Cells are Dispensable for NP Tissue Formation in an IVD‐Degenerative Mouse Model

**Figure 8:**
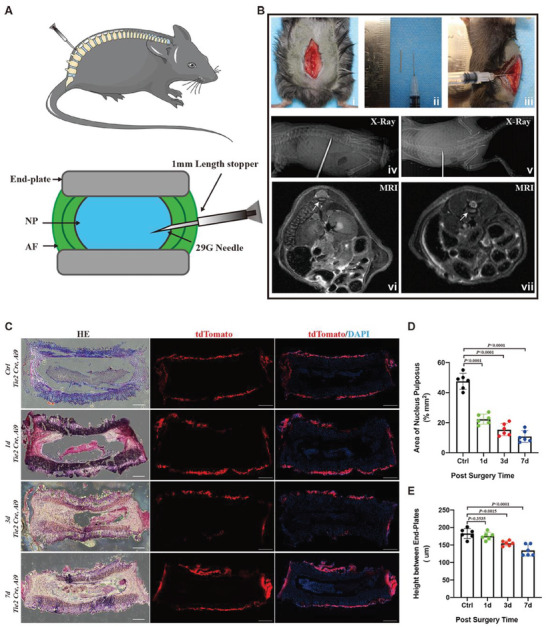
Tie2‐lineage cells do not contribute to NP tissue in an IVD‐degeneration mouse model. A) Schematic image of the experimental strategy for IVD needle puncture. NP tissues dissected from discs of mice cervical vertebra, thoracic vertebra, and lumbar vertebra by a homemade pick needle equipped with 1 mm stopper. B) Mice were anesthetized and the L3/L4, L4/L5, and L5/L6 discs were exposed with a dorsal longitudinal scalpel incision (i) under a 1 mm stopper (ii), then punctured with a homemade pick needle in lateral approach (iii). The features of the frontal and lateral X‐ray (iv, v) films and magnetic resonance imaging (MRI) (vi, vii) were validated effect. C) Representative immunofluorescence images of postoperative IVD sections (1d, 3d, 7d) from 4‐week‐old *Tie2‐Cre; Ai9* mice. D,E) NP region analysis showed the area of NP region decreased with postoperative time, and the height between endplates also changed with postoperative time. Values represent mean ± S.E.M., n.s. (not statistically significant) by an unpaired two‐tailed Student's *t*‐test. Scale bars, 200 µm (n = 6 mice per group).

While the above data demonstrated that Tie2+ cells did not label NP cells under physiological conditions, we established a surgically induced IVD‐degenerative mouse model^[^ ^43^ ^]^ (**Figure** **8**A,B). All the mice survived the surgical procedure and were resuscitated with no weight change over 1 week. The tissue integrity was then confirmed by H&E‐stained coronal sections, which showed that relative to that in the full IVD region, the size of the NP tissue area gradually decreased at 1, 3, and 7 days post puncture (Figure 8C,D). In addition, a statistical change in disc height or NP area was observed in the surgical groups compared to the control groups, which indicated that acupuncture in these models effectively disrupted the NP region in the IVD (Figure 8E). Similar to physiological conditions, Tie2‐driven tdTomato‐positive cells were detected only in the vertebral CEP and muscle area but not in the NP region at multiple postoperative time points under pathological conditions (Figure 8C). Taken together, multiple lines of evidence including spatial transcriptomic and genetic verification, reveal that Tie2 is not a true stem cell/progenitor marker for labeling NP‐derived cells, at least in a mouse model.

## 3 Discussion

With the application of scRNA‐seq technology to IVD biology, a series of single‐cell‐based transcriptional studies of candidate postnatal NPPCs have been performed in mice and humans.^[^ ^7a^ ^]^ However, these transcriptomic profiling approaches are limited not only by the lack of spatial information but also by the potential for the loss of fragile cell populations during collagenase digestion or cell sorting steps. In addition, physical tissue dissection may involve mixing non‐IVD cells into the final single‐cell database. To overcome these technical limitations, we used ST,^[^ ^10^, ^44^ ^]^ an emerging technique allowing spatially resolved transcriptional profiling of frozen histological sections of IVD tissue. To our knowledge, this represents the first spatial atlas of IVD gene expression.

Although the ST has the unique ability to discern local patterns of gene expression in IVD sections, there are still several notable limitations to this approach. First, even with optimized permeabilization, there is a balance between complete mRNA recovery and excess mRNA diffusion, which can disrupt the accuracy of spatial annotation. By determining the optimal time for tissue penetration, ST techniques can determine the point at which fluorescence signals are strongest, RNA diffusion is minimal, and the internal tissue structure is clearest. In this study, due to the distinctive matrix components present in different IVD units, the permeabilization conditions optimized here for the NP may not be completely suitable for the CEP and AF regions. Second, due to resolution limitations, each spatial spot in the 10x Genomics Visium platform represents multiple cells. This issue weakens the ability to transcriptionally distinguish adjacent or admixed cellular populations. For example, both Sp7 (Osterix) and Emcn (Endomucin) were detected in one spatial spot located in the CEP region due to the close proximity of osteoblasts and vascular endothelial cells, despite these transcripts being expressed in distinct cell types (data not shown). Third, whereas scRNA‐seq/bulk RNA‐seq methods sample the entire organs, current ST methods rely on histological tissue sections and therefore provide information in only 2D and only sample a subset of the organ.^[^ ^45^ ^]^ In the future, we anticipate the development of 3D ST technology that will allow for stereoscopic visualization of transcriptional profiles.

Using this first IVD ST atlas, we examined the spatial anatomical position of the candidate NPPC marker genes reported in prior studies (Figure 4). Interestingly, in the ST, postnatal NPPCs predominantly presented in the peripheral region of NP tissue differentiated toward the central region, which is consistent with the findings of recent studies in which lineage tracing tools such as *FGFR3‐creER* ^[^ ^7a^ ^]^ or *Uts2RcreER* ^[^ ^1^ ^]^ were used in vivo. Similarly, we herein evidenced that peripheral Ctsk‐expressing NP cells at the early postnatal stage indeed generated the whole morphological structure of the NP with IVD maturation. Our prior study showed that Ctsk lineage cells began to arise at postnatal day 0 in the NP but not in the CEP or AF. Taken together, these findings indicate that Ctsk is a new postnatal NPPC marker in addition to labeling periosteal stem cells and tendon progenitors; moreover, the fate and functions of Ctsk‐positive NPPCs need to be further investigated in pathological models of aging and disc herniation.

**Figure 9:**
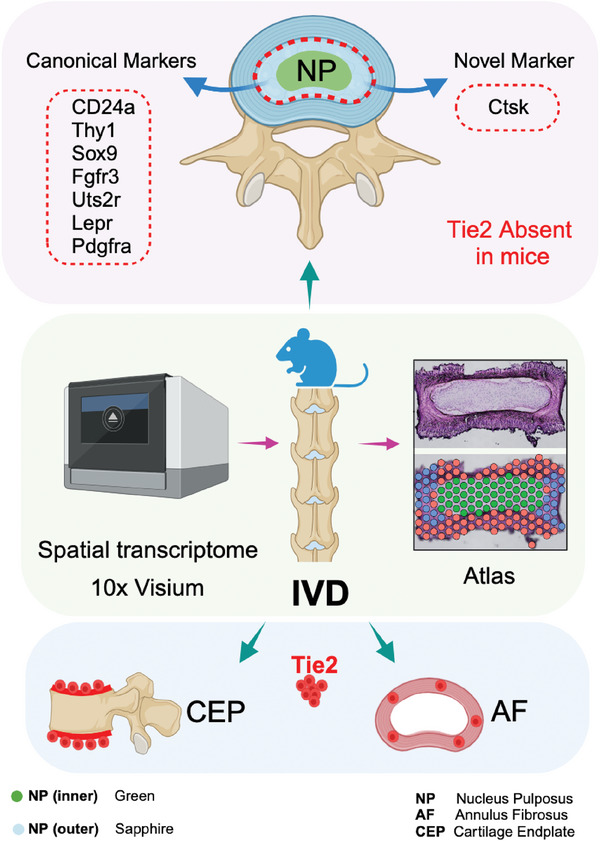
Graphical abstract showing various markers detected in the NP region, but Tie2 was expressed in the CEP or AF region but not NP.

The expression of Tie2, a cell‐surface marker suggested to label NPPCs, was absent in the NP region in this atlas of mouse IVD, a finding that was then further confirmed by ISS visualization. Cell lineage tracing is the gold standard and effective method for investigating the origin and fate of specific cell types in vivo. Consistent with the findings of the ST analysis, in vivo genetic evidence generated by using the *Tie2‐Cre* strain as a tracer further demonstrated that Tie2‐expressing cells and their descendants are vascular endothelial cells but not a physiological/pathological cellular sources of NP cells. In fact, in addition to its role in vascular endothelial cells, Tie2 is also expressed by other cell types, such as pericytes or a subset of macrophages. Prior reports of Tie2 presence in murine NP cells/tissues are likely due to potential contamination from these Tie2+ non‐NP cell types (**Figure** **9**, Graphical Abstract). Additionally, discrepancies are sometimes noted in stem cell marker levels between mice and humans are sometimes noted.^[^ ^46^ ^]^ Nonetheless, in the present study, we were still not able to exclude the presence of Tie2+ NPPCs in humans and nonmouse species. More future work needs to be conducted to enlarge our understanding of the functions of Tie2+ cells derived from the human IVD, especially to evaluate their ability to form NP‐like tissues in a stem cell‐based transplantation model in vivo, similar to the discovery of SSCs in humans.^[^ ^47^ ^]^

## 4 Experimental Section

### Clinical Specimens

This study protocol was approved by the Medical Ethics Committee of the First Affiliated Hospital of Sun Yat‐sen University (Guangzhou, China; No. [2022]033). All of the donors signed written informed consent for the use of the surgical technique in research on NP material. All the donors were informed about the purpose of the research, and no compensation was offered for donations. All twelve human NP tissue paraffin sections were carefully dissected from six donors in this study (Table S10, Supporting Information). The gelatinous tissue dissected from the central region was harvested as the Inner‐NP, and dissected from the peripheral region was harvested as the Outer‐NP.

### Animal Study

All mice used were maintained in a specific pathogen‐free environment. *Cdh5‐Cre, Rosa26‐tdTomato(Ai9)*, and *mTmG* mice were described previously.^[^ ^48^ ^]^ *Tie2‐Cre* mice, wild‐type (WT) C57BL/6J mice, *Pdgfra‐Cre* mice, *Ctsk‐GFP* mice, and *Ctsk‐CreER^T2^* mice were originally obtained from the Jackson Laboratory. WT mice at 3 weeks of age were used for the animal experiments in the present study. All animal experiments were conducted in accordance with the guidelines for housing and care of laboratory animals and performed following regulations approved by the Animal Care and Use Committee at Xiamen University (No. XMULAC20190084). *Ai9* mice were crossed with *Tie2‐Cre* mice, and *mTmG* mice were crossed with *Cdh5‐Cre* mice for multiple generations to generate homozygous *Tie2‐Cre; Ai9* and *Cdh5‐Cre; mTmG* mouse lines (Figure S1C, Supporting Information). Ai9 mice were crossed with *Pdgfra‐Cre* mice and *Ctsk‐CreER* ^T2^ mice to generate the *Pdgfra‐Cre; Ai9* mouse line and the *Ctsk‐CreER^T2^; Ai9* mouse line. Genotyping was performed with complementary DNA isolated from the paws (Figure S8A, Supporting Information). The primers, reaction components, and cycling conditions used were obtained from the Jackson Laboratory website (www.jax.org) (Table S11, Supporting Information).

### Cell Culture

To obtain mouse NP cells for *Tie2* detection, mouse NP tissues were harvested from the thoracic and lumbar intervertebral discs of 3w WT mice and cultured for 7 days in *α*‐MEM (Thermo Fisher Scientific, Waltham, MA, USA) supplemented with 10% fetal bovine serum (FBS) (Thermo Fisher Scientific) and Penicillin‐Streptomycin (Thermo Fisher Scientific) as described in previous publications.^[^ ^49^ ^]^

### Cell Sorting

NP tissue samples were harvested from Ctsk‐CreERT2; Ai9 mice, which were first dissected precisely and digested with collagenase for 6 h to disintegrate the tissues. Dissociated single NP cells were then stained with DAPI (1155MG010; BioFrox, Guangzhou, China) to label the dead cells. Flow cytometric analysis was performed with a BD AriaTM FUSION (BD, NJ, USA) at low temperatures and with a slow flow rate. Due to the vulnerability of NP cells, a low flow rate was maintained throughout the sorting process to reduce damage to cells by hydrodynamic pressure. The sorted cells were collected in a 15 mL centrifuge tube with *α*‐MEM containing 10% FBS to prevent the cells from sticking at 4 °C. NP cells were isolated and transferred to *α*‐MEM (HyClone) supplemented with 10% FBS and 1% penicillin/streptomycin solution on ice with a 1 mL pipette to reduce cell damage from aspiration pressure. The NP cells were sorted by FACS and suspended in Matrigel and plated into 48‐well plates. The flow cytometry data were analyzed using FlowJo X 10.0.7r2.

### In Vitro Multilineage Differentiation Assays

For osteogenic differentiation, the differentiation media used were as follows: 50 µg mL^−1^ ascorbic acid (Sigma) and 10 × 10^−3^ mm *β*‐glycerophosphate (Sigma). For adipogenic differentiation, the differentiation media used were as follows: 100 × 10^−3^ mm indomethacin (Sigma), 1 × 10^−3^ mm dexamethasone, 0.5 × 10^−3^ mm IBMX, 10 µg mL^−1^ human insulin and 1 × 10^−3^ mm rosiglitazone. For chondrogenic differentiation, the differentiation media used were as follows: 10 µg mL^−1^ insulin in combination with selenium, 10 ng mL^−1^ TGF‐*β*, 0.1 × 10^−3^ mm dexamethasone, 40 µg mL^−1^ proline, and 50 µg mL^−1^ ascorbic acid (Sigma).

### Stem/Progenitor Sphere Assay

For the stem/progenitor cell sphere assay, the prechilled culture surface of a 6‐well ultralow adherent dish (3471, Corning) was coated with a thin layer of Matrigel according to the recommended volume. The Matrigel was incubated with the gel for 30 min at 37 °C (not allowed to overwinter). A total of 2.4 × 10^5^ single‐sorted cells were resuspended in the appropriate volume of Matrigel on ice, and the mixture of cells and Matrigel was pipetted onto the precoated surface. Next, the Matrigel was incubated on the gel for 30 min at 37 °C (not allowed to overwinter), after which the recommended volume of culture medium was added. Agitation of the plate in the *x*‐*y* plane at intervals throughout 37 °C incubation might help prevent cell concentration in the center of the well (do not use a swirling motion, as cells would cluster around the well's edge). The remaining medium was chilled on ice, and 10% Matrigel was added. The Matrigel‐medium mixture was gently added to the plated culture. The cells were cultured and left undisturbed for 7 days, after which the Matrigel–medium mixture was added every 3 days. Images were taken with an Olympus fluorescence inverted microscope (CKX53) and analyzed with Cellview software (BI).

### Reverse Transcription and Real‐Time PCR

DNA‐free RNA was obtained using the Rneasy Plus Micro Kit (QIAGEN) with DNase treatment, and total RNA was reverse transcribed using a Hiscript II Q RT SuperMix for qPCR (+gDNA wiper) Kit (Vazyme, Nanjing, China). Real‐time PCR was performed in triplicate using ChamQ Universal SYBR qPCR Master Mix and the QuantStudio1 Real‐Time PCR System (Applied Biosystems, Foster City, CA) following the manufacturer's protocol. Gene expression was normalized relative to the gene expression levels of hypoxanthine guanine phosphoribosyl transferase (Hprt). The following primers are shown in Table S11 (Supporting Information).

### Spatial Transcriptomics (ST)

ST was performed using the Visium Spatial Gene Expression System (10x Genomics). 3w IVD tissues were harvested and stacked together, simultaneously fresh frozen in isopentane and subsequently embedded in OCT. The embedded tissue blocks were cryosectioned in a cryostat to generate appropriately sized sections for Visium Spatial slides while keeping the samples frozen. Sections were placed respectively on Visium Spatial Tissue Optimization Slide and a Visium Spatial Slide within the capture area. H&E staining and microscope brightfield imaging for sections were then processed. The section with the strongest fluorescence signal, minimum diffusion and longest time for permeabilization was chosen as the fit time for permeabilization. The cDNA libraries were sequenced on the Illumina sequencing platform by Genedenovo Biotechnology Co., Ltd (Guangzhou, China). The optimal permeabilization time (6 min) was used during library generation using a gene expression slide, with the first strand of cDNA synthesized via reverse transcription and the second strand of cDNA synthesized via PCR. Then, the cDNA was desaturated, disassociating the second strand of cDNA from the slide. The spatially barcoded, full‐length cDNA was amplified via PCR for library construction. Library construction, cleanup, and indexing were conducted with standard procedures. Read 1 and Read 2 were standard Illumina sequencing primer sites used in paired‐end sequencing. Samples were subjected to standard Illumina paired‐end sequencing using an Illumina HiSeq platform, which produced approximately two hundred million reads. Alignment and demultiplexing were conducted using the Space Ranger pipeline, and subsequent analyses were conducted using Seurat (version 4.2.0) in R. Before spot clustering, the data were normalized for more accurate clustering. To cluster the spots, modularity optimization techniques, such as SLM, were used to iteratively group the spots together, with the goal of optimizing the standard modularity function. The tSNE aims to place cells with similar local neighborhoods in high‐dimensional space together in low‐dimensional space. MAST^[^ ^50^ ^]^ (Model‐based Analysis of Single‐cell Transcriptomics) was used to determine the differential expression of a single cluster and generated a variogram^[^ ^51^ ^]^ to determine the differential expression among the spots in a section. To annotate the functions of the DEGs, GO term and KEGG pathway enrichment analyses of DEGs were performed using a self‐written program.^[^ ^52^ ^]^ Overall, the calculated p‐value was gone through FDR Correction, taking FDR of 0.05 as the threshold. Pathways meeting these criteria were defined as significantly enriched pathways in differentially expressed genes.

### RNA Velocity

RNA velocity analysis was performed through veloyto.py, velocyto.R.^[^ ^53^ ^]^ For this analysis, the spliced and unspliced reads were quantified by velocyto (version 0.17.17) with the human genome reference. The output loom file was analyzed for velocities of each gene following the pipeline of scvelo python package (version 0.1.19), and using scvelo_embedding_grid and velocity_embedding_stream function to visual differentiation trajectory onto the above UMAP in different subgroups.

### Amplification‐Based Single‐Molecule Fluorescence In Situ Hybridization (ASMFISH)

C57BL/6J WT mouse IVDs were dissected and fixed in 4% paraformaldehyde at 4 °C for 24 h, saturated with phosphate buffer‐saline containing 20% sucrose and 2% polyvinyl pyrrolidone with an additional 8% gelatin for overnight, embedded in OCT, and sectioned at 14 µm. AsmFISH probe hybridization and circularization, RCA and detection probe hybridization, and SNP genotyping assay were performed as previously described.^[^ ^54^ ^]^ All images in this study were exported as single channels.

### Histological and Immunofluorescence Analyses

Mouse IVD tissues were fixed with 4% paraformaldehyde at 4 °C for 24 h, saturated with phosphate buffer‐saline containing 20% sucrose and 2% polyvinyl pyrrolidone with an additional 8% gelatin for overnight, embedded in OCT, and sectioned at 6 and 20 µm. The H&E staining was performed on the 6‐mm‐thick IVD sections.^[^ ^55^ ^]^ Frozen sectioning and immunofluorescence staining were conducted according to a published protocol.^[^ ^56^ ^]^ The sections were incubated with primary antibodies against Osterix (Abcam, ab209484) followed by Alexa Fluor 647 donkey anti‐rabbit IgG H&L antibody (A11034, Invitrogen). The nuclei were stained with DAPI (A31573, Invitrogen). Images were acquired using a confocal microscope (TCS SP8 DLS, Leica, Germany) under identical imaging conditions using identical acquisition parameters, and fluorescence signals were quantified with Leica Application Suite X software (Leica).

### Immunohistochemistry

NP tissue paraffin sections were dewaxed and rehydrated, and heat‐mediated antigen retrieval was performed in the Ethylene Diamine Tetraacetic Acid (EDTA). The human NP paraffin sections were a kind gift from Prof. Peiqiang Su and Prof. Taifeng Zhou (The First Affiliated Hospital of Sun Yat‐sen University). To prevent nonspecific protein binding, endogenous peroxidase was treated for 10 min with 3% (v/v) hydrogen peroxide in methanol and 1% (w/v) bovine serum albumin (BSA). The sections were incubated with primary antibodies against CTSK (1:200, #ab19027; Abcam, Cambridge, UK) at 4 °C overnight. The samples were incubated with HRP‐conjugated secondary antibodies for 1 h at room temperature following the removal of extra primary antibodies, and the signals were detected with 3,3‐diaminobenzidine (DAB). The stained specimens were digitally photographed with a microscope (BX63; Olympus Corp., Tokyo, Japan).

### IVD Puncture Surgery

Needle punctures have been widely accepted as animal models for simulating the progression of IVD degeneration in vivo. IVD injuries were performed under general anesthesia (2% isoflurane in oxygen) and sterile conditions. Mice were anesthetized (3% isoflurane in oxygen) under an aseptic setting, and the surgery was fluoroscopically guided. Lumbar discs (L2‐3 and L3‐4) were identified by the anatomical position of the transverse vertebra and exposed with a 3 mm dorsolateral longitudinal incision and a 29 G (65% of disc height) needle was inserted into the discs through the NP center, controlling the damaged zone depth at 1 mm by a length stopper.^[^ ^43^ ^]^ Other discs that were not surgically exposed served as a sham control. The incisions were closed with sutures (Vicryl Rapide 4‐0 sutures; Ethicon, Somerville, NJ, USA). Mice were allowed free activity in cages and euthanized after 1, 3, and 7 days.

### Statistical Analysis

All the data were presented as the means ± SEs. Sample sizes were calculated on the assumption that a 30% difference in the parameters measured would be considered biologically significant with an estimate of sigma of 10%–20% of the expected mean. Unpaired, two‐tailed Student *t*‐tests were used for comparisons between the two groups. For multiple comparisons, one‐way analysis of variance (ANOVA) with the Bonferroni post hoc test was applied. The GraphPad PRISM software (version 5.0a; GraphPad, La Jolla, CA, USA) was used for data analysis. A P value less than 0.05 was considered to be statistically significant.

## Conflict of Interest

The authors declare no conflict of interest.

## Author Contributions

Y.C., L.Z., and X.S. contributed equally to this work and should be considered co‐first authors. R.X. designed and developed the experiments. Y.C., L.Z., and X.S. performed the main experiments and identified the genotype of genetic animals in this study. L.Z. and M.B.G. drafted the manuscript. J.C., J.H. and R.K. performed the bioinformatic analysis. X.Z., D.X., Z.L., C.Y., X.S., and N.L. helped perform the research. Z.C. and R.X. mentored the project and edited the manuscript. T.Z. and P.S. provide the research data for the manuscript. J.H. financed a part of the research data. All authors have read and agreed to the published version of the manuscript.

## Supporting information

Supporting Information

Supplemental Table 1

Supplemental Table 2

Supplemental Table 3

Supplemental Table 4

Supplemental Table 5

Supplemental Table 6

Supplemental Table 7

Supplemental Table 8

Supplemental Table 9

Supplemental Table 10

Supplemental Table 11

## Data Availability

The data that support the findings of this study are available from the corresponding author upon reasonable request.
