# Supplementary material for: Characterization of the Nucleus Pulposus Progenitor Cells via Spatial Transcriptomics
Source: Adv Sci (Weinh). 2024 Feb 4;11(18):2303752. doi: 10.1002/advs.202303752 (PMC11095158; doi:10.1002/advs.202303752)
Supplement: Supplementary file 1 — Supporting Information [file ADVS-11-2303752-s006.pdf]

ADVANCED SCIENCE
Open Access

Supporting Information

for *Adv. Sci.*, DOI 10.1002/advs.202303752

Characterization of the Nucleus Pulposus Progenitor Cells via Spatial Transcriptomics

Yu Chen, Long Zhang, Xueqing Shi, Jie Han, Jingyu Chen, Xinya Zhang, Danlin Xie, Zan Li, Xing Niu, Lijie Chen, Chaoyong Yang, Xiujie Sun, Taifeng Zhou, Peiqiang Su, Na Li, Matthew B. Greenblatt, Rongqin Ke, Jianming Huang*, Zhe-Sheng Chen* and Ren Xu**

Supporting Information

No. advs.202303752R1

Title: Characterization of the nucleus pulposus progenitor cells in spatial transcriptomics

Running title: Nucleus pulposus progenitor cells in a spatially resolved transcriptional atlas of the intervertebral disc

Authors: Yu Chen[1, 2*], Long Zhang[1,2*], Xueqing Shi[1,2*], Jie Han[1,2], Jingyu Chen[3], Xinya Zhang[4], Danlin Xie[4,5], Zan Li[1,2], Xing Niu[6], Lijie Chen[6], Chaoyong Yang[7], Xiujie Sun[8], Taifeng Zhou[9], Peiqiang Su[9], Na Li[1,2], Matthew B. Greenblatt[10,11], Rongqin Ke[4#], Jianming Huang[12#], Zhe-Sheng Chen[13#] and Ren Xu[1, 2#]

Contact information:

[1] The First Affiliated Hospital of Xiamen University-ICMRS Collaborating Center for Skeletal Stem Cells, State Key Laboratory of Cellular Stress Biology, Faculty of Medicine and Life Sciences, School of Medicine, Xiamen University, Xiamen, 361102, China.

[2] Xiamen Key Laboratory of Regeneration Medicine, Fujian Provincial Key Laboratory of Organ and Tissue Regeneration, School of Medicine, Xiamen University, Xiamen, 361102, China

[3] Gene Denovo Biotechnology Co, Guangzhou 510006, China.

[4] School of Medicine and School of Biomedical Sciences, Huaqiao University, Quanzhou 362000, China

[5] School of Life Sciences, Westlake University, Hangzhou, 310030, China

[6] China Medical University, Shenyang 110122, Liaoning, China.

[7] Department of Chemical Biology, College of Chemistry and Chemical Engineering, Xiamen University, 361005, China

[8] Department of Obstetrics and Gynecology, Xiang'an Hospital of Xiamen University, School of Medicine, Xiamen University, 361102, China

[9] Department of Spine Surgery, Guangdong Provincial Key Laboratory of Orthopedics and Traumatology, The First Affiliated Hospital of Sun Yat-sen

University, Guangzhou 510080, China.

[10] Department of Pathology and Laboratory Medicine, Weill Cornell Medical College, New York, NY 10065, USA.

[11] Research Division, Hospital for Special Surgery, New York, NY 10065, USA.

[12] Department of Orthopedics, Chengong Hospital (the 73th Group Military Hospital of People's Liberation Army) affiliated to Xiamen University, Xiamen 361000, China.

[13] College of Pharmacy and Health Sciences, St. John's University, New York, NY 11439, USA.

*These authors contributed equally to this work and should be considered co-first authors.

#Address all correspondence and requests for reprints to:

Ren Xu

State Key Laboratory of Cellular Stress Biology,

School of Medicine, Xiamen University,

A503, Yuejin Building, Xiang'an South Road, Xiang'an District, Xiamen 361102, China (xuren526@xmu.edu.cn)

Zhe-Sheng Chen

College of Pharmacy and Health Sciences, St. John's University,

8000 Utopia Parkway, Queens, New York, NY 11439, USA (chenz@stjohns.edu)

Jianming Huang

Department of Othopedics, the 73th Group Military Hospital of PLA, NO.94, Wenyuan Rd., Siming District, Xiamen City 361003, Fujian, China (8303260@qq.com)

Rongqin Ke

School of Medicine and School of Biomedical Sciences, Huaqiao University,

269 Chenghua North Road, Fengze District, Quanzhou 362021, China (rke@hqu.edu.cn)

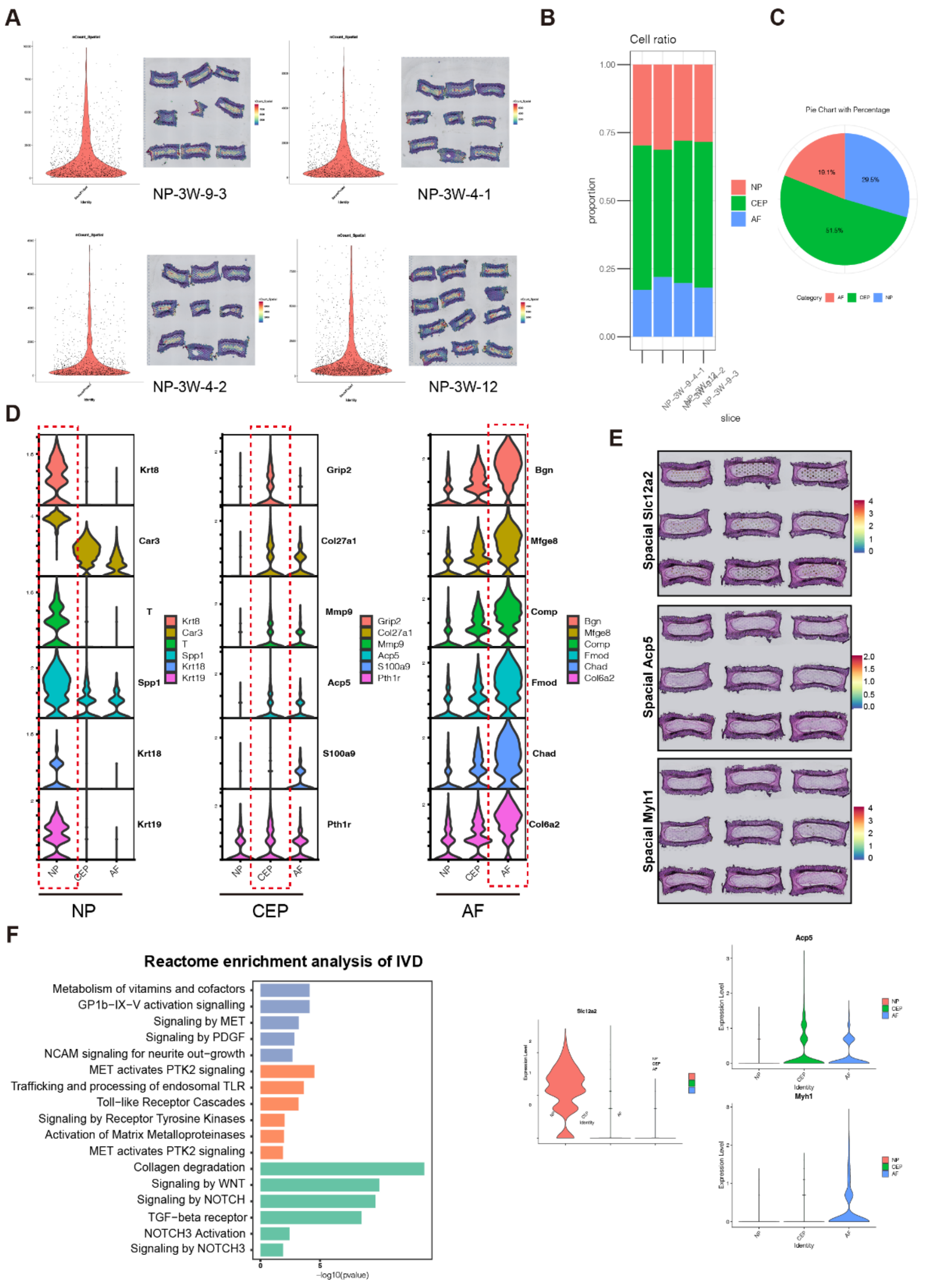

Figure S1: A-C The ST data quality control of 4 chips. **D** Violin plots showing the expression levels of representative genes in NP, CEP and NP cell subclusters. **E** Violin plots showing the expression levels of Slc12a2, Acp5 and Myh1 genes in NP, CEP and NP cell subclusters, respectively. **F** The reactome analysis in IVD.

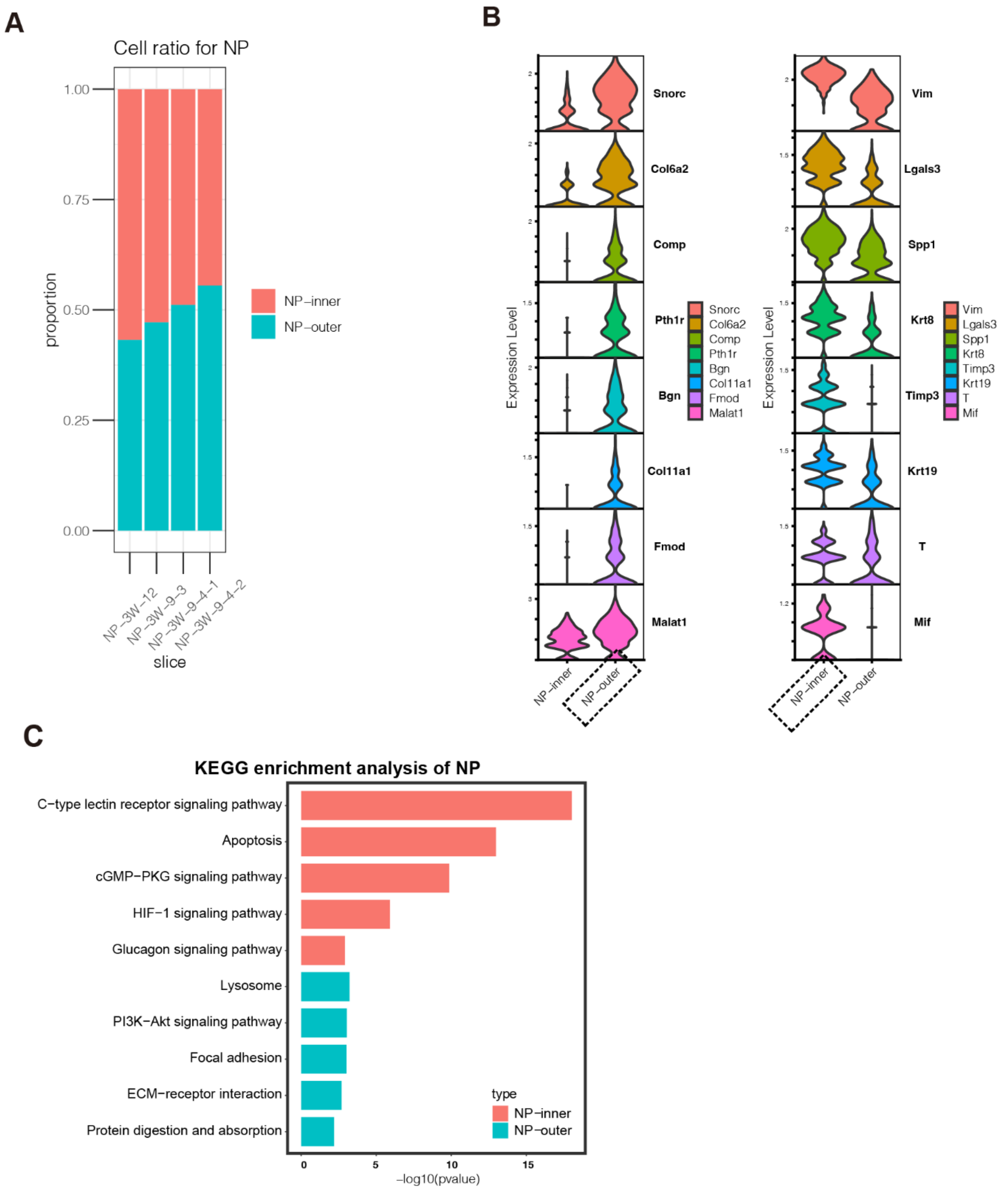

Figure S2: **A** The ST data quality control of 4 chips. **B** Violin plots showing the expression levels of representative genes in NP cell subclusters. **C** KEGG analysis of the DEGs showing the enriched signaling pathways in NP.

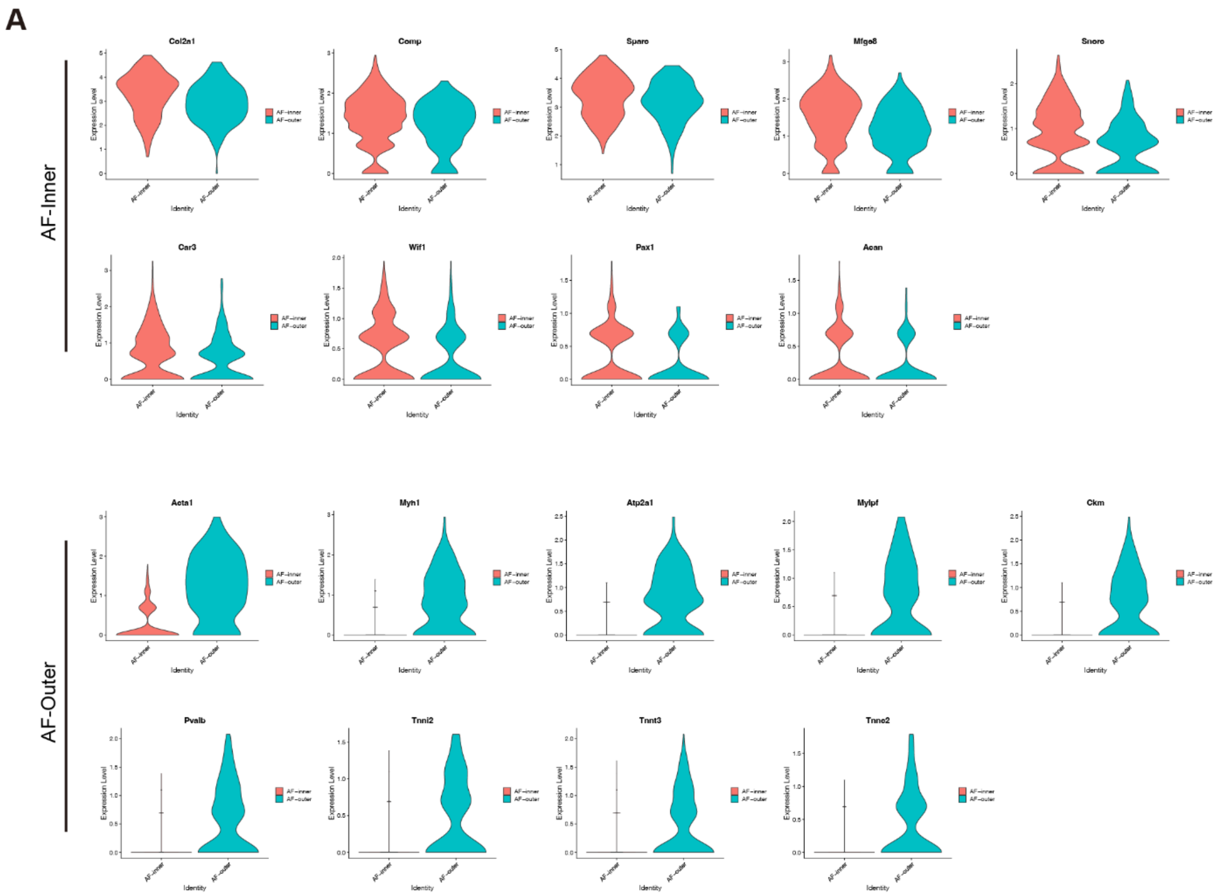

Figure S3: A Violin plots showing the expression levels of representative genes in AF-inner cluster and AF-outer cluster.

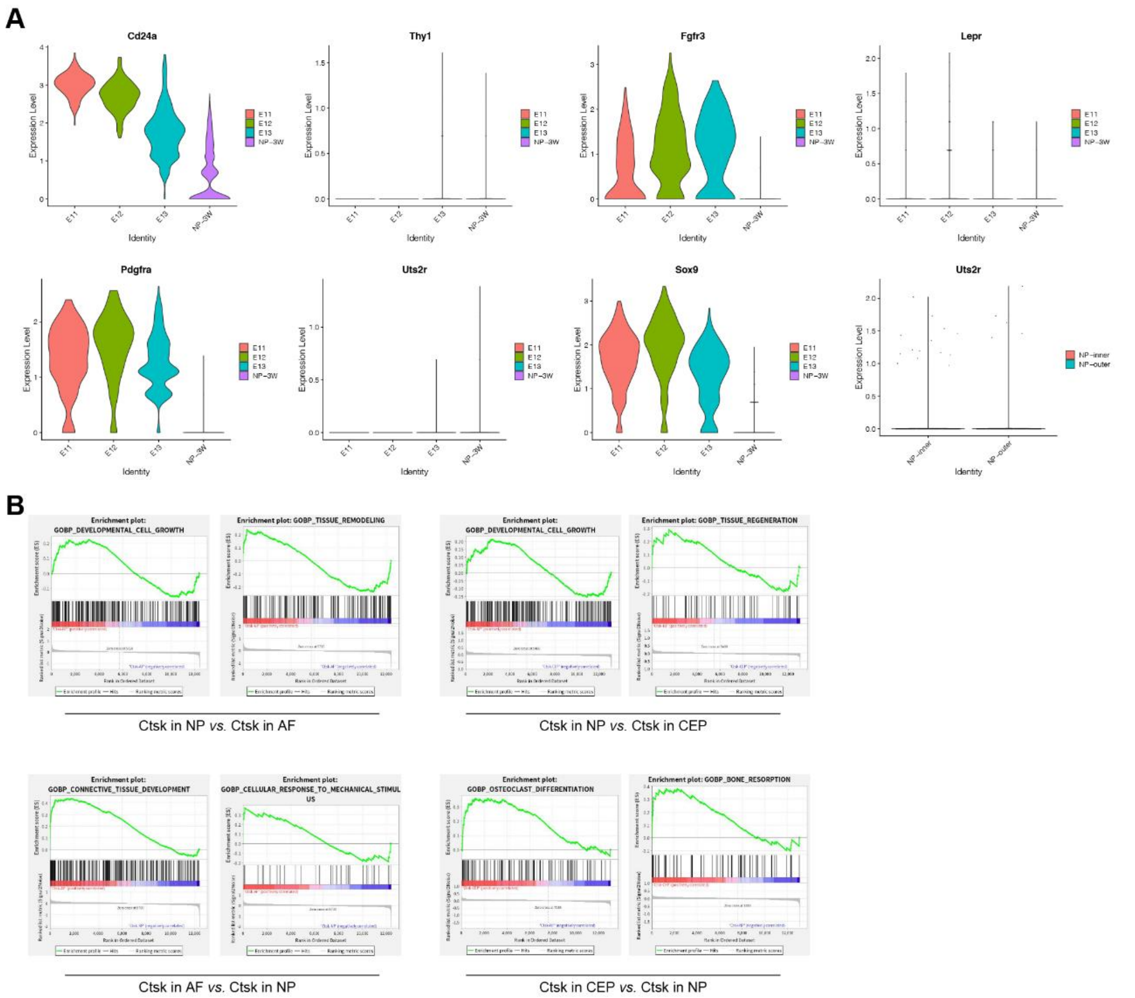

Figure S4: A Violin plots showing the expression levels of CD24a, Thy1, Sox9, Fgfr3, Uts2r, Lepr, Tie2 and Pdgfra at the indicated developmental stages. **B** GSEA showing the enrichment of function between groups (Left top, Ctsk in NP vs Ctsk in AF; Right top, Ctsk in NP vs Ctsk in CEP; Left bottom, Ctsk in AF vs Ctsk in NP; Right bottom, Ctsk in CEP vs Ctsk in NP).

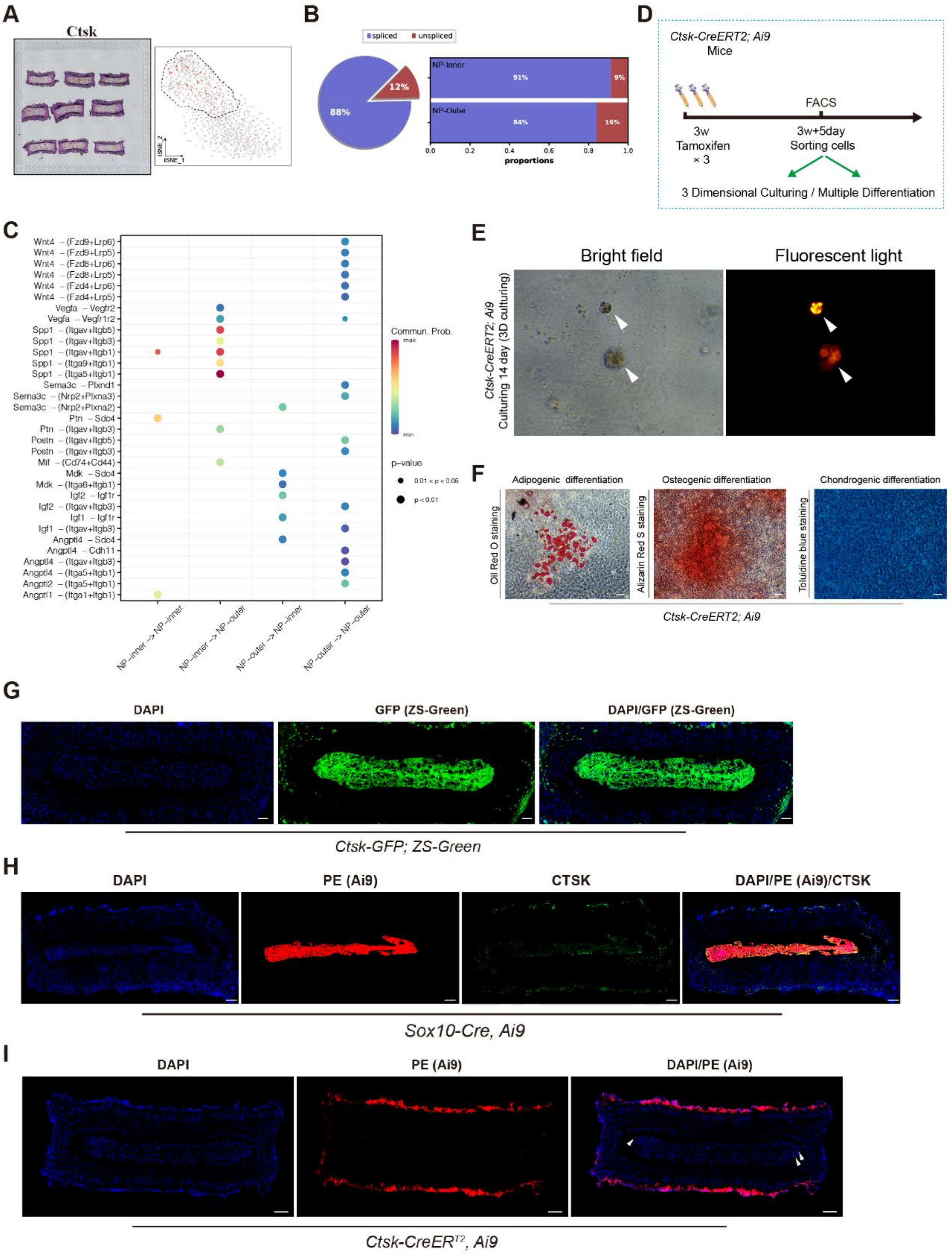

Figure S5: A Spatial feature plot and the tSNE plots analysis of Ctsk in the NP region. **B** The spliced and unspliced percentage of RNA velocity. **C** Dot plots showing the signals contributing mostly to outgoing or incoming signaling of NP cell groups. **D** Schematic image of the experimental strategy for cell culturing. **E** Three-dimensional culturing of Ctsk+ cells. **F** Multiple differentiation of Ctsk+ cells. **G** Ctsk+ cells in IVD of the *Ctsk-cre; ZS-Green*

mice at 3 weeks. **H** Ctsk+ cells in IVD of the *Sox10-cre; Ai9* mice. **I** Ctsk+ cells in IVD of the *Ctsk-CreERT2; Ai9* mice injected with Tamoxifen at 3 weeks and harvested after 24 hours.

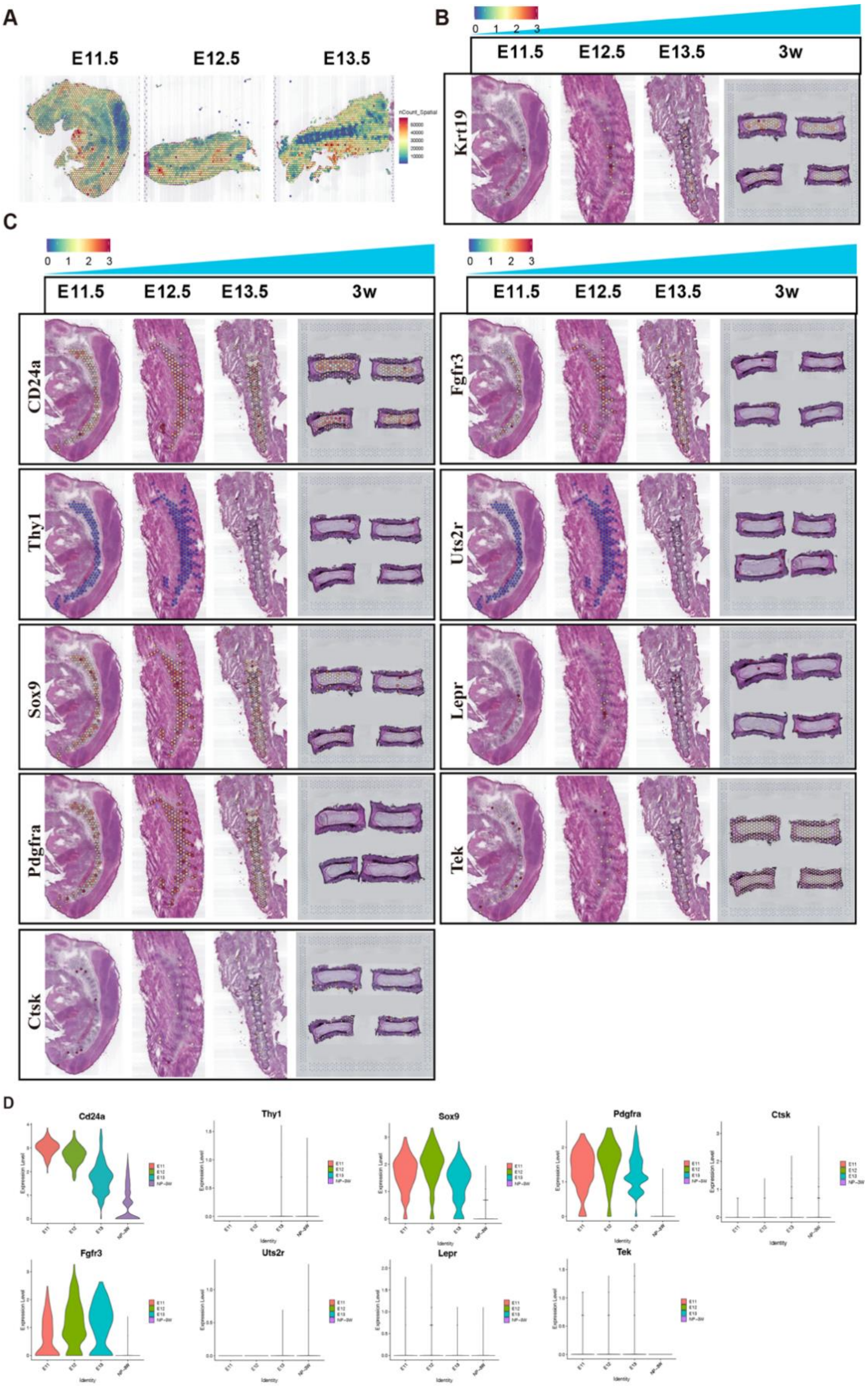

Figure S6: A Projection of spots including defined mouse IVD cells on the histological section at E11.5, E12.5 and E13.5. **B** Visualization of Krt19 expression on the histological sections at the indicated developmental stages. **C** Visualization of CD24a, Thy1, Sox9, Fgfr3, Uts2r, Lepr, Pdgfra, Tie2 and

Ctsk expression on the histological sections at the indicated developmental stages. **D** Violin plots showing the expression levels of maekers abovementioned at the indicated developmental stages.

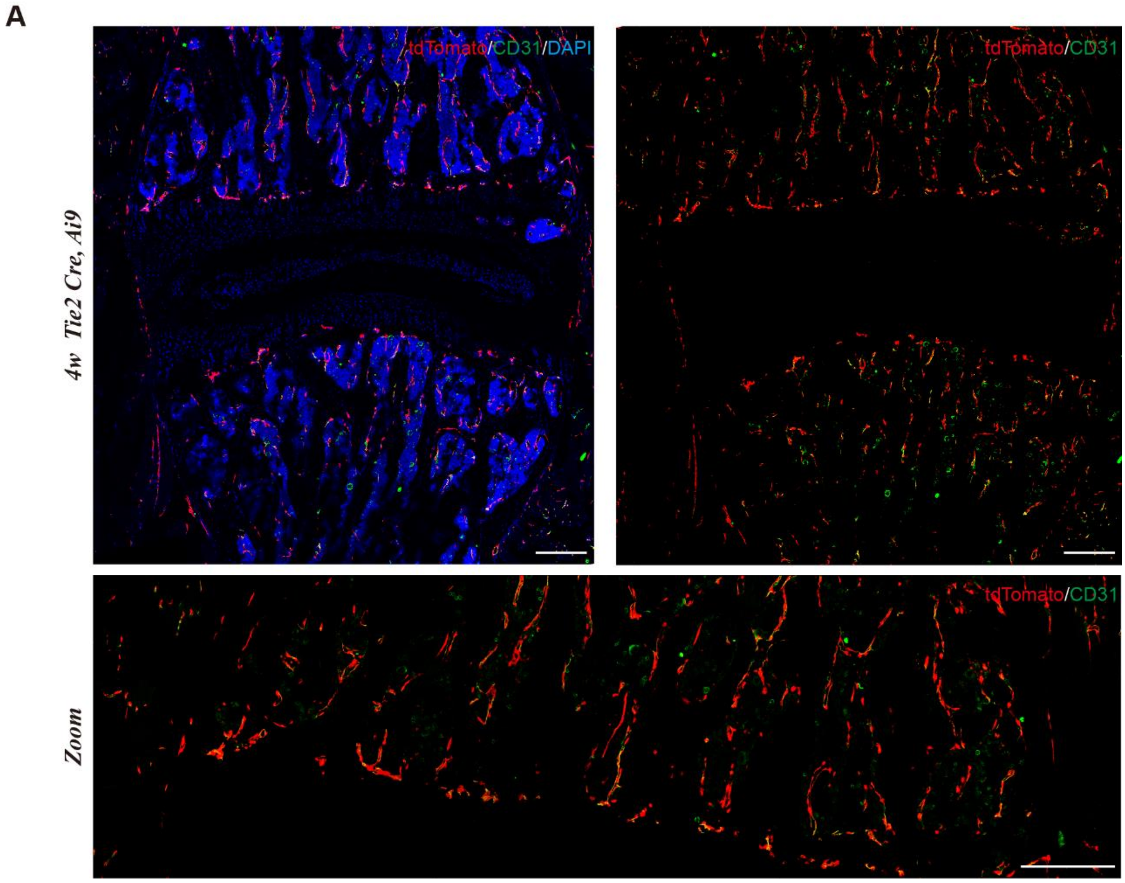

Figure S7: A Representative image of Tie2 and CD31 staining from *Tie2-Cre; Ai9* mice (red) stained for DAPI.

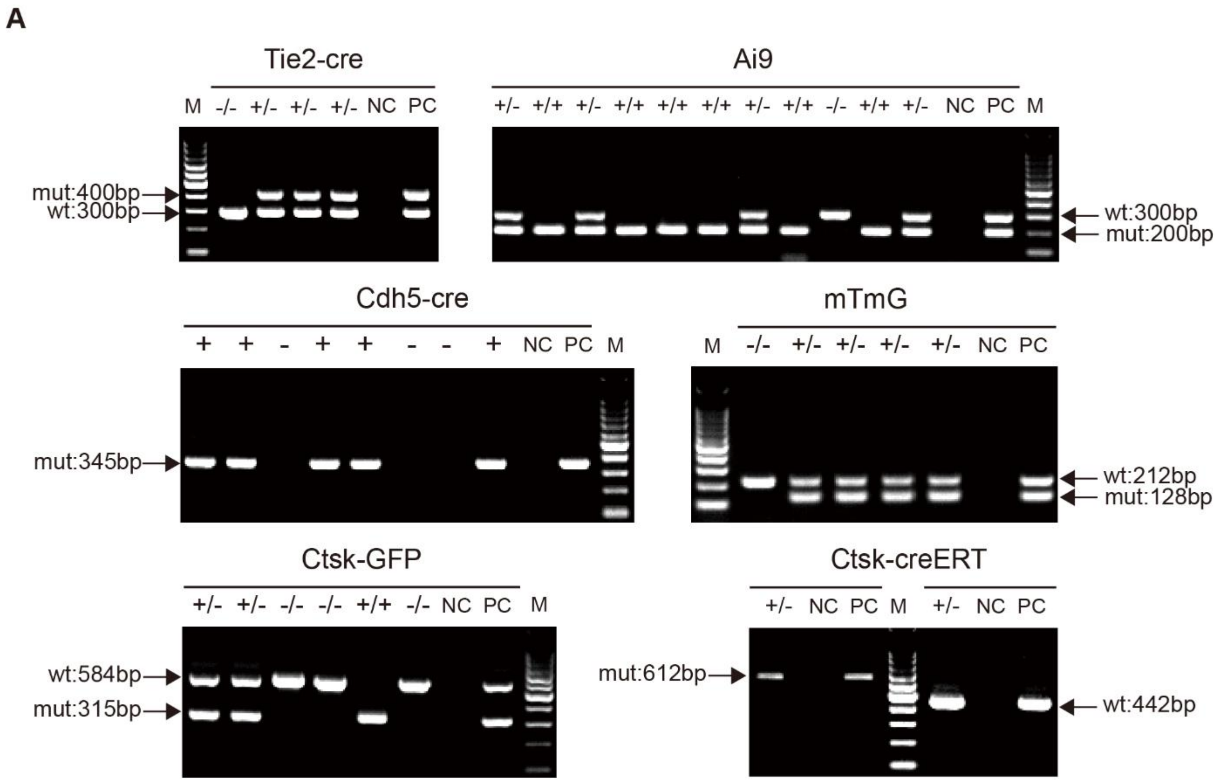

Figure S8: A The banks of genotyping used in this study.
